# Navigating the micro-politics of major system change: The implementation of Sustainability Transformation Partnerships in the English health and care system

**DOI:** 10.1177/13558196221142237

**Published:** 2022-12-14

**Authors:** Justin Waring, Simon Bishop, Georgia Black, Jenelle M Clarke, Mark Exworthy, Naomi J Fulop, Jean Hartley, Angus Ramsay, Bridget Roe

**Affiliations:** 1 Health Services Management Centre, 1724 University of Birmingham, UK; 2 Business School, 6123 University of Nottingham, UK; 3 Wolfson Centre for Population Health, Queen Mary, University of London, London, UK; 4 Dept of Applied Health Research, 4919 University College, London, UK; 5 School of Social Policy, Sociology and Social Research University of Kent, UK

**Keywords:** major system change, micro-politics, political skills

## Abstract

**Objective:**

To investigate how health and care leaders navigate the micro-politics of major system change (MSC) as manifest in the formulation and implementation of Sustainability and Transformation Partnerships (STPs) in the English National Health Service (NHS).

**Methods:**

A comparative qualitative case study of three STPs carried out between 2018–2021. Data collection comprised 72 semi-structured interviews with STP leaders and stakeholders; 49h of observations of STP executive meetings, management teams and thematic committees, and documentary sources. Interpretative analysis involved developing individual and cross case reports to understand the ‘disagreements, ‘people and interests’ and the ‘skills, behaviours and practice’.

**Findings:**

Three linked political fault-lines underpinned the micro-politics of formulating and implementing STPs: differences in meaning and value, perceptions of winners and losers, and structural differences in power and influence. In managing these issues, STP leaders engaged in a range of complementary strategies to understand and reconcile meanings, appraise and manage risks and benefits, and to redress longstanding power imbalances, as well as those related to their own ambiguous position.

**Conclusion:**

Given the lack of formal authority and breadth of system change, navigating the micro-politics of MSC requires political skills in listening and engagement, strategic appraisal of the political landscape and effective negotiation and consensus-building.

## Introduction

Major system change (MSC) has become a prominent feature of contemporary health care reform.^1,2^ This typically involves reconfiguring how multiple care organisations are coordinated and work together within a given service area or locality to promote integration, efficient use of scarce resources and aggregate health benefits.^1^ System change is often complicated by disagreements about the re-allocation of roles, resources and relationships,^3^ and how such disagreements are managed leads to variability in the implementation of change.^4^ Research suggests that system leaders need to be effective at managing competing preferences and interests, although Jones et al.^5^ find that research too often presents a ‘technicist view’ that neglects the complex micro-politics of system change. We show that many of these micro-political challenges relate to leaders’ lack of formal authority in the care system and of being positioned between the governance structures of ‘sovereign’ health and care organisations, which together mean that leaders need to focus on building consensus for change through the voluntary participation of multiple (often competing) stakeholders.^2–4,6^

This paper focuses on the micro-politics of formulating and implementing Sustainability and Transformation Partnerships (STPs) in the English National Health Service (NHS). Unlike other cases of MSC that tend to focus on relatively discrete service areas, such as stroke, cancer or major trauma,^3,4,6^ STPs represent broader programmes of system change aimed at promoting service integration within defined regions across England.^7^ Sustainability and Transformation Partnerships build on a long history of attempts to promote integration of health and social care in the English NHS,^8^ which has involved experimentation with Integrated Care Pilots (later ‘Pioneers’) and New Care Models (‘Vanguards’). Forty-four STPs were introduced across the English NHS in 2016 addressing priorities in primary care, prevention, early intervention, mental health, productivity and workforce development. STPs eventually evolved into regional Integrated Care Systems (ICSs) in which NHS organisations, local government and other agencies work together to provide integrated ‘place-based’ care,^9^ and which became statutory bodies in July 2022.^10^ These reforms reflect a continuing emphasis on place-based reorganisation of care with corresponding changes in the commissioning and provision of health and social care services.^11^

Integrating care, especially at scale, can be difficult^4^ and is frequently hampered by the incompatibilities between professional values, governance arrangements and funding systems.^12^ This study investigated how health and social care leaders understand and navigate the micro-politics of MSC as found in the formulation and implementation of STPs. It develops two lines of analysis. First, it describes the prominent political issues experienced in the formulation and implementation of STPs, where the focus is primarily on the strategic arena of planning and governance. Second, it analyses the strategies and behaviours of those leading STPs as they try to resolve these political issues.

### A micro-politics perspective on MSC

A developed body of critical interpretative scholarship characterises health care services as complex political arenas that are in many ways defined by disagreements and struggles.^13–15^ Many studies have analysed how the managerialisation and marketisation of care have prompted micro-political turmoil as health care professionals negotiate and resist change regarded as threatening their professional interests.^13,16^ Although these studies offer rich empirical accounts of the strategies deployed by managers and professionals to influence change, they tend to explain such micro-politics in terms of deeper macro-political tensions around structural interests, especially the relative power of professions vis-à-vis the neoliberal state. Reflecting on the politics of quality improvement, Langley and Denis^17^ suggest change is often complicated by the micro-politics of competing value systems, the distribution of benefits, and the relative opportunities for stakeholders to influence change processes. However, such studies rarely offer a developed conceptualisation of the political strategies and skills of those leading (or resisting) change.

In contrast, research within the field of management studies more directly describes the political strategies and skills of interpersonal influence.^18–21^ The concept of political skill is commonly used to characterise a person’s ‘ability to effectively understand others at work, and use this understanding to influence others to act in ways that enhances one’s personal and/or organizational objectives’.^21(p.291)^ A growing body of health services research has applied this concept to understand how health care leaders understand and mediate competing perspectives when implementing change.^22^ Montalvo and Byrne^23^ argue that nurse leaders can better motivate others, foster teamwork and manage conflict through the use of political skills. Rogers et al.^24^ also show that managers’ use of political skills can mediate competing interests and foster a shared sense of purpose in multi-disciplinary teamwork. Waring et al.^25^ describe five aspects of health care leaders’ political skills and behaviour when implementing change: ‘personal and interpersonal qualities’ relating to self-belief and resilience; ‘strategic thinking’ relating to the ability to understand the political landscape; ‘communication skills’ for engaging and influencing stakeholders; ‘networking skills’ to build connections between stakeholders and ‘relational tactics’ for dealing with difficult individuals. While these ideas more directly attend to the strategies, tactics and skills of micro-politics, they often focus on individual behaviour to the neglect of deeper structural interests and ideologies.^22^

Reflecting on these perspectives, there is scope to more fully account for the political skills and behaviours of leading system change, whilst remaining attentive the ways such politics is shaped by structural interests and ideologies. Developing this view, we make two observations relevant to the implementation of MSC. First, research has tended to focus on the micro-politics of *intra*-organisational change, rather than *inter*-organisational change. We suggest that a distinct set of tensions characterises large-scale integration initiatives^12^ and, significantly, these are often played out in the spaces *between* the formal authority and governance structures of health and social care organisations.^3^ Second, research has often focused on the micro-politics of health care marketisation, rather than more collaborative forms of organising.^2^ Although there are clear points of overlap, these modes of governance reflect different ideological imperatives that might alter the terms on which micro-politics is played out. Reflecting on these observations, it might be expected that those leading the implementation of MSC need to use a distinct set of political skills and behaviours to mediate competing preferences and engender collaboration in the absence of formal authority.

Informed by the above literature, this study investigated how health and social care leaders understand and navigate the micro-politics of MSC as found in the formulation and implementation of STPs. It examined, first, the political issues experienced in the strategic formulation and configuration of change; and second, the political strategies, skills and behaviours used by those leading STPs as they seek to deal with these political issues.

## Methods

We carried out qualitative case study research with three STPs between Summer 2018 and Spring 2021. The paper reports on the relatively high-level strategic formulation and implementation of each STP, rather than the operational delivery of specific change projects.

### Case selection and recruitment

We carried out a desk review of all 44 STPs to identify similarities and differences in spatial-demographic profile, health and social care system and strategic priorities. Candidate cases were identified to investigate variations across these areas, with particular attention to the interplay of the number, size and specialism of care commissioners and provider organisations, the urban and rural demographic profile in terms of number and size of cities, towns and population diversity, and the thematic priorities for change. Candidate STPs were contacted in writing and following engagement activities, three STPs were recruited.

Table 1 presents a summary overview of key features of each STP. Case A (Willow) comprised three small-to-medium sized general hospitals, two community hospitals, one ambulance service, one commissioner and one local authority, which operated across three medium-sized towns; Willow also bordered Greater London. Case B (Elm) was made up of one medium-sized hospital, one large mental health and community care provider, one large teaching hospital, one ambulance service, multiple commissioner organisations and two local authorities with a focal medium-sized city. Case C (Oak) comprised two small-to-medium-sized community rural hospitals, one medium city-based hospital, one ambulance provider, one merged commissioner and one local authority based around a single medium-sized city and large rural area.

**Table 1.** Summary features of participating sustainability and transformation partnerships.

	Case 1 willow	Case 2 elm	Case 3 oak
Population	1.2 million	1.1 million	I Million
Geographical footprint	Three medium-sized towns (c150,000 people) and villages, on edge of greater London	One medium-sized city (c350,000), several towns and rural villages	One medium-sized city (c250,000), several towns large settlements (c100, 000) and numerous smaller villages
NHS trusts	6 incl. Ambulance	4 incl. Ambulance	5 incl. Ambulance
Commissioning bodies	1 merged clinical commissioning group (CCG)	6 clinical commissioning group (CCG) then 1 merged CCG	1 merged clinical commissioning group (CCG)
Other care services	Community health social care enterprise	Community health social care enterprise	Private community interest company
Local authorities	1	2	2
Strategic objectives	• Working more collaboratively as a system	• Organising care around individuals and populations	• Moving from boundaries to people and communities
	• Prevention, early intervention, and tackling wider determinants of health	• Prevention and health education	• Addressing system issues
	• Planning services some across larger areas and some more local	• Long-term conditions	• Contracts and commissioning
	• Collaborate across support functions	• Help people remain independent	• Placed based care
	• Designing ‘system architecture’ to support governance and decision-making	• Support and provide care in the community (health and social)	• Central role of primary care
	• Agreeing quality standards	• Integrated care	
		• Minimise variation	
Participants	26, including	32, including	14, including
	• Sustainability and transformational partnership (STP) chair	• STP director	• STP director
	• Transformation lead	• Chief executive local authority	• STP deputy director
	• Chief executive of NHS trust	• Transformation lead	• STP managers (2)
	• NHS trust representative for integration	• NHS trust representatives	• CCG representatives (2)
	• Chief executive, local authority	• NHS trust medical director	• NHS trust representative
	• Local authority lead for integration	• Lead for urgent care	• Medical director community NHS trust
	• Lead for integration	• Lead for mental health services	• Lead for workforce development
	• Lead for medicines	• Local authority lead for adult social care	• Lead for organisational development
	• Lead for women and children	• CCG lead	• Lead for estates and facilities
	• Lead for maternity services	• CCG representatives (3)	• Lead for cancer
	• Lead for community pharmacy	• Police services representatives	• Project managers (2)
	• Medical lead for acute medicine	• Major trauma representatives	
	• Medical lead for family medicine	• GP representatives (9)	
	• General practitioner (GP) representatives (2)	• Project manager (3)	
	• Healthwatch representatives	• Communications lead (2)	
	• Community group representative	• Personal budget lead	
	• CCG lead	• Voluntary sector representative	
	• CCG representatives (2)	• Community representative (2)	
	• Programme managers (3)		
	• Project managers (3)		

Note: STP – sustainability and transformation partnerships; CCG – clinical commissioning group; GP – general practitioner.

### Data collection

Semi-structured interviews were the primary method of data collection. Recruitment was based on a participant’s involvement in the governance or operation of each STP. They were identified and recruited through a review of public documentation, snowball sampling with gatekeepers, and observations of meetings. In total, 72 people participated in 83 interviews across the three STPs (Willow *n* = 26, Elm *n* = 32, Oak *n* = 14; see Table 1 for summary of roles). Using a topic guide, the interviews explored experiences of disagreements in the development of each STP, the people involved, and the strategies used to resolve these.

Field researchers also carried out non-participant observations of senior leadership meetings, management teams, thematic committee meetings and project team meetings, which were recorded in field journals. In total, 28 meetings were observed over 49 h (Willow 22 h, Elm 22 h, Oak 5 h). Many people participated in informal ‘in situ’ conversations to clarify observations. As a result of the COVID-19 pandemic, almost all observations with the Oak STP were carried out online, which facilitated accessibility but limited exposure to interpersonal dynamics.^26^ Two focus groups were carried out with this STP to deepen data collection. A large volume of documentary sources was collected for each STP to provide contextual understanding and inform sampling including: strategy documents, public online information, organograms and information videos.

### Data analysis

Data analysis was undertaken through manual and computer-assisted coding. Case reports were developed for each STP, including (i) a narrative account of each case; (ii) a detailed account of the defining disagreements and (iii) interpretations relevant to the study objectives. Cross-case analysis focused on understanding the ‘*disagreements*’*, people and interests’* and the ‘*skills, behaviours and practic*e’. For the purpose of this study, disagreements and disputes were identified from situations where a plurality of preferences, agendas or interests were articulated by social actors, and where the resolution of these disputes was necessary for coordinated, consensus-based decision-making. The latter stages of analysis engaged with the existing literature on the micro-politics of health care reform, especially Langley and Denis^17^ and Waring et al.^25^

## Findings

We first describe first the micro-politics of formulating and implementing STPs, and second, the political strategies, skills and behaviours of managing these micro-political issues.

There were marked micro-political differences between cases. Willow (Case A) comprised three small-to-medium sized general hospitals, which served relatively discrete communities and which had some history of collaboration but limited strategic coordination, leading to some engrained tensions. Elm (Case B) was characterised by one large teaching hospitals that had significant influence on the wider health care system relative to others care providers, as well as multiple commissioning organisations who, until they merged, struggled to tackle the influence of this NHS Trust. And Oak (Case C) had one medium-sized acute NHS Trust based in the city, but whose influence in the wider rural area was limited. As such, the configuration and history of each system shaped the specific disputes and issues confronted in the formulation and operation of the STPs, in common with other examples of MSC.^1^ While each STP was, thus, defined by micro-political struggles, we identified six common issues that provided the focus of such politics, where representatives from the NHS, local authorities, voluntary sector care providers, private enterprise and community groups tried to influence the formulation and implementation of system change (Table 2).

**Table 2.** The micro-political issues of formulating and implementing STPs.

Political issue	Descriptive	Underlying factors driving politics
Politics of purpose	Disputes about local health needs, the problems of the care system and the potential for system change	Differing philosophies of care
		Differing meanings and values of ‘integration’
		Differing meanings and values of ‘the system’
Politics of governance	Disputes about STP leadership and coordination, in terms of who hold roles and how they are enacted	Different views about the legitimacy of system leaders with the absence formal or statutory authority
		Differing preference about the approach taken to system governance, planning and management (directive vs facilitative)
		Different views about whose interests are served by system leaders
Politics of inclusion	Disputes around the extent of inclusion and representation in leadership and decision-making	Differing meanings and values of inclusion and democratic representation
		Differing preferences about the approaches taken engagement and deliberation
		Questions about the dominance of certain groups and the peripheral involvement of others
Politics of system architecture	Disputes about the redistribution of functional roles, responsibilities and resources	Differing preferences to maintain or challenge the status quo linked to threat to symbolic/functional status
		Differing interpretations of fairness, risk and benefit
		Differing preferences for new models of integrated working
		Questions about the processes of decision-making and the extent of representative involvement
Politics of resource sharing	Disputes about the sharing of scarce financial, human, technological and physical resources that are linked to established models of care organisation	Underlying threats to control of key resources and symbolic/functional status
		Differing preferences about the control of resource and decision-making processes
		Differing interpretations of fairness, risk and benefit
		Underlying threats to control of key resources and symbolic/functional status
Politics of prioritisation	Disputes about what transformation projects should take priority to resolve system issues	Differing interpretations of need
		Different expectations around evidence and data
		Different expectations about decision-making processes

Although these political issues could be seen as relatively discrete, they strongly intersected as part of a wider political struggle; for example, where disagreements about governance were tied to disagreements about resource sharing. There was some degree of sequential order in how issues progressed in that they unfolded overtime as a series of linked events, but it was common for ‘downstream’ disagreements to re-ignite previously resolved disputes. Our study examined how those leading the STPs worked to resolve these political issues, or at least manage them with sufficiency to afford progress in the functioning of the STP, while acknowledging that in some instances disagreements led to periods of hiatus and instability. Here, we focus on three mutually constituted political fault-lines that appeared to underpin the observed micro-politics, while maintaining a focus on the political strategies used by system leaders to resolve these.

### Meaning and values associated with ‘integration’

Many disagreements centred on underlying differences in meaning and values associated with the broad project of ‘integration’. Although stakeholders ostensibly shared a broad commitment to more integrated working, there were underlying differences in the cultural and ideological outlooks of professions, organisations and care sectors. These were often manifest in how they gave meaning to (i) notions of ‘care’, and health and wellbeing; (ii) the rationale and purpose of ‘integration’ as a model of care and (iii) ‘the system’ as entity through which integrated health and social care is delivered.

These differences were observed in the development of the Elm STP’s ‘prevention and early intervention’ strategy. Representatives from the community health, public health and social care sectors emphasised the importance of promoting healthier lives, managing long-term health conditions, and tackling health disparities. Integration was interpreted as a way for multiple agencies to collaborate in achieving these goals through the redesign of person- and community-centred services. Those from the acute hospital sector were not ignorant to these ideals, but from their perpective the main value of such a service was to reduce inappropriate demand for episodic (curative) care. Integration was viewed as a means of rebalancing responsibilities across primary, community and social care services.

[A]nd it really quickly became apparent that nobody was on the same page, you know, there was no agreement about what the priorities were. *(Project Lead, Elm)*

Such differences were reflected in how stakeholders talked about the regional care ‘system’ as the focus for integration. For some, the system was envisioned as multiple services that could be better coordinated, while for others, the system was described as a geographical place comprising communities and people with particular needs.

In seeking to foster integration, STPs leaders needed to reconcile such differences or at least find ways for productive co-existence. Almost all leaders talked about the importance of listening to the diverse viewpoints of regional actors to find common agendas. Various listening and engagement methods were observed, from one-to-one meetings to ‘town hall’ meetings. These not only helped STP leaders better understand the points of convergence and divergence, but they also had a performative quality for demonstrating their willingness to be inclusive of multiple perspectives.

It’s about being inclusive… so people can feel engaged and inspired….I know how difficult it can be to try to force people, doctors, to accept change and it has to feel that it is driven by the community. *(Field interview with STP Director, Oak)*

In trying to find points of meaningful alignment and mutual understanding, STP leaders talked of facilitating dialogue between opposing groups and adopting a conflict resolution role. By hearing each other’s viewpoints, stakeholders could identify mutual interests which engendered collaborative working. We observed, for example, how leaders carefully stage-managed interactions so that there was a focus on shared concerns, whilst downplaying historical disagreements. This was encapsulated by the idea of ‘seeing the big picture’ and creating an STP that improves the lives of local people. It seemed difficult for stakeholders to openly argue against this goal.

They might think system change is important in their hearts but not necessarily in their heads. *(STP Director, Elm)*

We have got to remind people why we are doing this. The people of [place] deserve a better care service. *(Field interview, STP Deputy Director, Oak)*

In other settings, we observed how STP leaders would openly challenge groups taking a highly partial view. In these interactions, those resistant to change were often singled out for collective scrutiny. Through such engagement strategies, the STP leaders did not necessarily resolve the deeper differences between stakeholders, but they crafted an overarching vision that stakeholders could broadly align with or from which outliers could be identified.

### Perceptions of ‘winners and losers’ in system change

Many disagreements centred on how stakeholders perceived the reconfiguration of roles, responsibilities and resources as creating ‘winners and losers’. For some, change was viewed favourably because it reflected their preferred way of working, increased access to resources or elevated their status, but others viewed the same initiative in diametrically opposite terms. Given the importance of maintaining stakeholders’ commitment to the project of integration, STP leaders used a number of strategies to influence the perception of winners and losers and to mitigate the perception of (particularly financial) risk.

[We are] supposed to be working together, but there will still be the financial directors of organisations that will want to maintain their books and balance the books. So there is still that financial tension which has an impact, as well as workload issues that get passed across. *(GP representative, Oak)*

To illustrate, a proposal for a new community mental health service within the Elm STP was viewed by service user groups as offering people greater choice in meeting their wellbeing needs, while voluntary sector providers saw it as an opportunity to expand their role in service provision. In contrast, representatives from the region’s mental health provider questioned the clinical merits of this proposal and even though they recognised the benefits of services that helped people avoid inpatient care, they were opposed to re-allocating financial resources to ‘un-evidenced’ interventions. As such, project leaders had to persuade specialists of both the clinical benefits of new services, and that the re-allocation of funds might have longer term benefits in reducing demand.

We know [it] work[s] for other communities, and we really need to find the evidence that they can work for people with personality disorder. We need to show the outcomes for people can be better and that there are potential savings to the system. *(Field note, Personal Care Budget Lead, Elm)*

STP leaders used a number of strategies for managing the winners and losers of system change. The first was to appraise the likely impact of change and anticipated the responses of stakeholders. This seemed to be based upon a combination of strategic foresight, that is, a realistic assessment of change and learning from past experiences. By working out ‘what matters’ to stakeholders and how they might react, STP leaders were better placed to promote or ‘sell’ change in ways that would foster support and reduce opposition. It also helped with identifying potential allies who could act as earlier adopters and then champion the benefits of change to more reticent groups.

We have been working with [Team Name] for a few years now as part of our community out-reach programmes. They have helped us organise and deliver events and they have helped us get the message out in ways that probably makes more sense to the people of [place]. *(Project Manager, Willow)*

The second strategy centred on dealing with those stakeholders who saw themselves as losing out, but where their on-going involvement was needed for the delivery of new services or the sharing of resources. For example, the Elm STP experienced deadlock when the local authority social care services threatened to withhold their support for a new community-based rehabilitation services if a particular service provider was not given a major role in leading the project. STP leaders confronted such opposition in a number of ways. As above, a primary strategy was to influence how stakeholders interpreted change by promoting the value of system working. This often centred on a utilitarian vision in which all contributed something to realise the broader goals of improved community health, and this might involve short-term loses for long-term gains. A common line of rhetoric that leaders used in both public and private interactions was that ‘*everyone gives up a little*’ and the system gains a lot.

[Y]ou can talk about how, well if we can work better together as a group and you may lose a little bit and we may lose a little bit. We work together and in an altruistic way it’s better for patients, it might be more efficient, some losers, some gainers, that kind of approach, give and take approach, and our negotiations can occur in that fashion. *(GP Representative, Elm)*

A related strategy was to engage stakeholders in negotiation and deal-making whereby perceived loses were traded or off-set with gains or compensation in other areas. One prominent strategy was to offer new leadership roles in system change as a form of compensation for agreeing to new resource sharing arrangements or giving up areas of their work.

It’s so important to find the things that will keep them interested, it doesn’t always have to be more resource or pandering to their egos, it is better when it’s about the issues that really matter to the service and communities. *(Lead for Urgent Care, Elm)*

### Structural differences in power and Influence

The micro-politics of system change was shaped by underlying lines of power and influence. In broad terms, the prevailing lines of power placed some regional stakeholders in more influential or central positions, while marginalising others. As noted above, within the Elm case the large teaching hospital was recognised as influential in the region because of its profile of specialist providers; whereas in the Oak case, the larger city-based general hospital was seen as having more influence on the healthcare system than the smaller rural-based hospitals. However, such positions varied according to the particular service or population health issues; in general terms, those with access to or control over certain resources, with enhanced professional or organisational status or, more often, with specialist expertise, were typically more influential or privileged in the social organisation of care. The significance of these power dynamics for the STPs was that those with most influence, for example, specialist care providers, could be more reticent about systems working, because of the potential threat to their status or control over resources. In contrast, those with least influence or status, for example, social care and voluntary sector providers, were often most enthusiastic about system change because it could enhance their role and status. As such, some STP leaders talked about readdressing power imbalances as a way to foster better integration.

A significant complicating factor was that those leading the STPs had a relatively ambiguous and, at times, disputed position within prevailing governance structures. Although each STP created relative hierarchical governance systems, comprising executive boards, management groups and thematic committees, it was widely noted that these structures lacked statutory authority and functioned outside pre-existing governance arrangements of ‘sovereign’ health and care organisations.

We are in a precarious position. We are a small team and we don’t really fit anywhere, but we are responsible for making sure the plans are taken forward. *(Field notes: STP Transformation Lead, Oak)*

In addressing their precarious position, STP leaders often talked about enhancing their credibility within the existing lines of power, working constructively with stakeholders and demonstrating their functional contributions. Across all three STPs, leaders adopted a facilitative and non-directive style, so that their role was not to tell people what to do, but to help them work better together. A number of leaders talked of presenting a diplomatic or neutral style so that they operated above the established lines of power and worked to mediate conflict and promote harmony. Taking these approaches seemed important for establishing the symbolic position of STP leadership based on consent in the absence of formal authority.

It’s just building that rapport up with the person that’s leading the project and I suppose it’s about telling them … you know, because everything’s to do with performance reporting or performance management, you’ve got to take out the fact that I’m not there to beat them with a stick, you know it’s beneficial for me and the person running the project to get the information that I need to help them run that project. *(Project Manager for Women’s and Children’s Services, Willow)*

However, the precarious position of STP leaders remained a constant challenge and necessitated the use of political influence and persuasion in the absence of formal authority. STP leaders described the need to ‘read the landscape’ and understand the prevailing lines of power. Some talked about developing a type of political ‘radar’ to work out who were the key people, what interests are at stake, and what tactics they might use. Many gave examples of professional figureheads or professional cliques who opposed change, and the capacity for organisations to withhold resources or opt-out because they did not support change.

You do need to be able to recognise when people are using their political skill to their own ends and potentially negatively and I think that’s something that you need your sort of antennae out. *(Transformation Lead, Elm)*

STP leaders described a range of relational strategies for managing the informal lines of power. Some described fostering positive relationships with influential leaders with the aim of gaining intelligence about likely opposition and, at the same time, finding areas for negotiation or constructive engagement.

The key challenge is to actually understand why people that you interact with are doing what they’re doing and to really try to get to know what their drivers are, what are the things that cause them anxiety every day. *(Programme Manager, Elm)*

Many talked about the need to build alliances, especially amongst peripheral or marginal groups with less influence, who could then collectively counteract the more powerful groups who oppose change.

[S]ome of it’s been about developing relationships and alliances… getting a better, clearer, shared understanding across the different stakeholders about the current situation and what the facts are and what the evidence is, and what needs to change. *(Project Manager for Integrated, Willow)*

Some described directly challenging stakeholders who purposefully undermined change. These participants tended to have relatively senior positions within their own organisation or a significant professional reputational within the local care system which provided a strong basis to confront those who opposed change.

Don’t be afraid to sometimes challenge the status quo. Now that’s hard and dependent on what level, well depending on how senior you are and what your relationships are like, sometimes that’s easier said than done and I think for a lot of staff. *(STP Director, Oak)*

## Discussion

The study found that the formulation and implementation of each STPs was, in many ways, defined by micro-politics. This was manifest in disagreements among stakeholder representatives from health and social care, voluntary sector, private enterprise and community groups, where each sought to advance or protect their particular preferences, agendas or interests through engaging in political strategies and behaviours. This is similar to the findings of Fulop et al.^4^ on the reconfiguration of regional stroke services. Although their study did not explicitly deal with the micro-politics of change, it showed how the resolution of disagreements at key stages in the implementation journey resulted in different service models and care outcomes. Where the Fulop et al. model presents a relatively step-wise implementation journey, our study finds that political disagreements are more dynamic, recursive and interconnected so the resolution of one issue has implications for others.

Through our analysis we aimed to look beyond the overt manifestations of disagreement to identify the more fundamental tensions that drive the micro-politics of system change^5^ especially health and social care integration.^12^ Following Langley and Denis,^17^ we suggest that the micro-politics of system change is grounded in differences in meaning and values, perceptions of and reactions to potential gain and loss, and underlying lines of power and influence. Furthermore, we suggest that these dimensions often overlap, have similar triggers and are mutually constituted, especially where meanings of integration are closed linked to perceptions of benefits and positions of influence. In discrete, but also overlapping ways, these dimensions condition the types of political strategies and tactics used by those leading system change as they work to reconcile diverse meanings, find acceptable distributions of benefits and risks, and navigate established lines of power.

When dealing with diverse meanings, leaders draw upon a range of communicative skills, especially the ability to listen and understand different viewpoints, to downplay difference and find shared points of agreement, and to influence how stakeholders made sense of system change through appeals to seeing the, big picture. Such framing strategies have been shown in other cases of system redesign where leaders use expertise, evidence and public voice to construct compelling cases for change.^27^ It has also been shown that where incompatible perspectives undermine collaboration, leaders can construct parallel frames that influence multiple groups.^28^

When reconfiguring health and social care systems, leaders need to calculate how change will be perceived as creating ‘winners and losers’, and how such perceptions will influence on-going engagement. Our study supports the idea that system leaders need to ‘attend to history’^1,29^ to understand how past conflicts shape future change. Our study also demonstrates the importance of leaders’ negotiation and deal-maker skills. As shown by Black et al.,^30^ system change often provokes a sense of loss and, therefore, managing this and maintain stakeholder involvement is key to on-going integration efforts. This can include, for example, finding inducements or forms of compensation to off-set perceived losses.^3^ Our findings demonstrate that this is a core strategy employed in STP micro-politics.

The underlying lines of power and influence present arguably the most significant challenge.^13^ Our study finds that in the absence of formal authority, STP leaders used a range of strategies for dealing with influential groups and establishing their own position, which echo recent research on health care leaders’ political skills.^25^ Interestingly, much of this relied on interpersonal tactics that address the more localised expressions of power, rather than the deeper lines of structural power.^18^ That is, leaders rarely challenged the elevated power of all doctors, but they worked with key medical representatives to find ways of overcoming localised resistance.^16^ This also involved cultivating networks of marginal or less powerful groups to counteract the influence of dominant actors. Significantly, our study finds that these system leaders are themselves often disadvantaged by the prevailing lines of power, especially as they lacked formal statutory powers at the time our study was conducted. As such, their efforts to formulate and implement system change are largely dependent on their politics skills to influence, persuade and negotiate amongst competing interests.

The types of political strategies and skills observed in our study broadly reinforce the emerging literature.^25^ In particular, they closely resemble the types of strategic thinking, communication skills, networking skills and inter-personal tactics described by Waring et al.^25^ However, our study shows how these might vary at the system level, where the need to foster inter-organisational and inter-sectoral working, often in the absence of formal authority, exacerbates the types of challenges associated with implementing organisational change. This includes, for example, the diversity of value systems around care and integration, the complex redistribution of roles and resources and multiple cross-cutting lines of power.

Returning to the literature on the micro-politics of health care organisation, our study seeks a middle ground that does not reduce analysis to either structural interests or behavioural competencies. As noted above, the prevailing literature either takes a more critical and structural view that explains micro-politics in terms of macro-political interest,^14,15^ or presents a relatively behavioural view of leaders’ political competencies and skills.^20,24^ Our study retains attention to both perspectives and, importantly, shows the connections between them through examining the types of behaviours and skills used by leaders when confronting local manifestations of macro-political tension in meaning, rewards and power. Our study shows how the individual strategies and behaviours are both conditioned by and directed towards resolving deeper political fault-lines.

Our research offers important contributions to understanding the role of system leaders in MSC and integrated of care systems, more broadly. Fulop et al.’s^4^ study of stroke reconfiguration showed how leadership styles can impact the implementation of system change. Specifically, where leaders were found to ‘hold the line’ in the face of opposition, rather than engaging in consultation, they established a model of care that more closely aligned with the national guidelines and resulted in improved care outcomes. In a related study, Turner et al.^31^ suggested that a combination of leadership styles is often needed whereby designated leaders set the strategic direction and keep stakeholders engaged, while distributed leadership supports on-going involvement in change. Taking a designated or directive approach is arguably more feasible when supported by formal authority, although this does not negate the need for engaged and distributed forms of leadership. However, where formal authority is lacking and where the breadth of change is more diverse, as in the case of the STPs, it seems difficult for those leading change to take a directive or authoritative approach, at least all of the time. Rather, negotiation and consensus-building become the customary basis for fostering consent and integrated working, hence the need for political skills. Recent legislation has given ICSs (the newly reconstituted STPs) in England a clearer statutory foundation in the form of Integrated Care Boards that now hold formal responsibility for commissioning community-wide health and social care services. That said, it is unlikely that the allocation of formal authority will resolve the underlying political tensions described in this study, and so leaders will continue to need political strategies and skills as they create more integrated models of care.
